# Supplementary material for: Weakly supervised classification of aortic valve malformations using unlabeled cardiac MRI sequences
Source: Nat Commun. 2019 Jul 15;10:3111. doi: 10.1038/s41467-019-11012-3 (PMC6629670; doi:10.1038/s41467-019-11012-3)
Supplement: Supplementary file 3 — Description of Additional Supplementary Files [file 41467_2019_11012_MOESM3_ESM.docx]

**Description of Additional Supplementary Files**

**File Name: Supplementary Movie 1**

**Description:** Tricuspid Aortic Valve (TAV), example MRI sequence with velocity encoding (VENC).

**File Name: Supplementary Movie 2**

**Description:** Tricuspid Aortic Valve (TAV), example MRI sequence with raw encoding (CINE).

**File Name: Supplementary Movie 3**

**Description:** Tricuspid Aortic Valve (TAV), example MRI sequence with magnitude encoding (MAG).

**File Name: Supplementary Movie 4**

**Description:** Bicuspid Aortic Valve (BAV), example MRI sequence with velocity encoding (VENC).

**File Name: Supplementary Movie 5**

**Description:** Bicuspid Aortic Valve (BAV), example MRI sequence with raw encoding (CINE).

**File Name: Supplementary Movie 6**

**Description:** Bicuspid Aortic Valve (BAV), example MRI sequence with magnitude encoding (MAG).
